# Dietary Protein Restriction during F_0_ Pregnancy in Rats Induces Transgenerational Changes in the Hepatic Transcriptome in Female Offspring

**DOI:** 10.1371/journal.pone.0021668

**Published:** 2011-07-07

**Authors:** Samuel P. Hoile, Karen A. Lillycrop, Nicola A. Thomas, Mark A. Hanson, Graham C. Burdge

**Affiliations:** 1 Academic Unit of Human Development and Health, Faculty of Medicine, University of Southampton, Hampshire, United Kingdom; 2 Development and Cell Biology, Faculty of Natural and Environmental Sciences, University of Southampton, Southampton, Hampshire, United Kingdom; Victor Chang Cardiac Research Institute, Australia

## Abstract

There is considerable evidence for non-genomic transmission between generations of phenotypes induced by environmental exposures during development, although the mechanism is poorly understood. We investigated whether alterations in expression of the liver transcriptome induced in F1 offspring by feeding F0 dams a protein-restricted (PR) diet during pregnancy were passed with or without further change to two subsequent generations. The number of genes that differed between adult female offspring of F0 protein-restricted (PR) and protein-sufficient (PS) dams was F1 1,684 genes, F2 1,680 and F3 2,062. 63/113 genes that were altered in all three generations showed directionally opposite differences between generations. There was a trend toward increased proportions of up-regulated genes in F3 compared to F1. KEGG analysis showed that only the Adherens Junctions pathway was altered in all three generations. PR offspring showed altered fasting glucose homeostasis and changes in phosphoenolpyruvate carboxykinase promoter methylation and expression in all three generations. These findings show that dietary challenge during F0 pregnancy induced altered gene expression in all three generations, but relatively few genes showed transmission of altered expression between generations. For the majority of altered genes, these changes were not found in all generations, including some genes that were changed in F3 but not F1, or the direction and magnitude of difference between PR and PS differed between generations. Such variation may reflect differences between generations in the signals received by the fetus from the mother as a consequence of changes in the interaction between her phenotype and the environment.

## Introduction

Variations in the quality of the early life environment can induce, through epigenetic changes, multiple phenotypes from a single genotype. In humans, environmental constraint during development, such as poor nutrition, is associated with increased risk of non-communicable diseases in later life including the metabolic syndrome and cardiovascular disease [1]. In a number of animal models, the offspring of mothers given poor nutrition during pregnancy exhibit pathophysiological changes similar to human disease [1]. For example, maternal dietary protein restriction in rats induces hypertension, dyslipidaemia and impaired glucose tolerance in the adult offspring [2]. Furthermore, there are a number of examples from natural history in which the environment experienced by the mother induces an altered phenotype in the offspring [3].

There is now considerable evidence for non-genomic transmission of induced phenotypic traits between generations [4]. Such processes may provide an important mechanism for the inheritance of disease traits in humans [5], [6]. For example, mortality from diabetes was increased in men if the paternal grandfather had been exposed to abundant nutrition during their pre-pubertal slow growth period [7]. Also, the daughters of women exposed to nutrient restriction and stress during pregnancy as a result of the Dutch Hunger Winter showed decreased birth weight and an increased risk of insulin resistance, and, in turn, their granddaughters also were born with a lower birth weight despite adequate nutrition during their daughter's pregnancy [8], [9]. Similarly, metabolic dysregulation induced by nutritional or hormonal interventions during F0 pregnancy in animal models can be transmitted to more than one generation of offspring. For example, feeding pregnant rats a protein-restricted (PR) diet during pregnancy in the F0 generation induced elevated blood pressure and endothelial dysfunction [10], and insulin resistance [11], [12] in the F1 and F2 offspring, despite adequate nutrition following weaning and during pregnancy in the F1 generation. Adverse effects of feeding a PR diet during F0 pregnancy on glucose homeostasis have been detected in F3 offspring [13]. Finally, the administration of dexamethasone to dams in late pregnancy induced an increased expression of the glucocorticoid receptor (GR) and of its target gene phosphoenolpyruvate carboxykinase (PEPCK) in the liver of the F1 and F2 offspring, although these effects were lost in F3 offspring [14].

Although the passage of induced traits between generations is well known, the underlying mechanisms are poorly understood. Epigenetic processes have been suggested to be involved [6]. Supplementation of the diet of F0 A^vy^ mice with methyl donors induced phenotypic changes through altered methylation of the A^vy^ locus in F1 and F2 offspring [15]. Hypomethylation and increased mRNA expression of the hepatic PPARα and GR promoters has been reported in F1 and F2 offspring of F0 dams fed a PR diet during pregnancy [15]. Female germ cells which give rise to the F2 generation are formed in F1 offspring when they were *in utero* and thus are exposed to the environmental challenge to which the F1 offspring are exposed. Thus it is unclear whether the results of these studies represent transmission of induced phenotypes between generations. Therefore, conclusive demonstration of such transmission between generations requires that induced changes are detected in F3 offspring [17]. Anway *et al.* [18] showed transmission essentially without further change of altered male fertility and changes in promoter methylation of specific genes in testis up to F4 generation offspring of F0 dams exposed to the endocrine disruptor vinclozolin. Such germline effects involved altered expression of DNA methyltransferases [19].

Most previous reports of transmission of altered phenotypes between generations have focused on the mRNA expression of specific candidate genes [14], [16]. However, Skinner *et al.* reported transcriptome-wide changes in the hippocampus and amygdale of F3 offspring of F0 rats which received the endocrine disruptor vinclozolin during pregnancy [20]. These findings showed altered expression of 92 genes in the hippocampus and 276 genes in the amygdale in F3 males and of 1,301 genes in the hippocampus and 172 genes in the amygdale in F3 females. However, the expression of the transcriptomes of these tissues in was not reported in F1 and F2 offspring. This raises the question of whether the same changes are induced in the transcriptome in every subsequent generation of offspring following an insult during F1 development, even when there is no challenge during development in those subsequent generations. Information about the expression of the transcriptome in successive generations may therefore provide important insights into the mechanism by which induced traits are transmitted between generations. For example, if the same alterations in mRNA expression were present in F1, F2 and F3 offspring, this would suggest transmission of induced traits unchanged between generations, possibly through germ cells. However, differences in the number, type or direction of changes in the transcriptome between generations would suggest adjustments in the regulation of transcription in each generation. Such variation may involve changes in the signals received by the fetus as a result of differences in in the interaction between the phenotype of the mother and the environment in each generation. In the present study, we investigated the effect of feeding a PR diet to F0 dams during pregnancy on the expression of the liver transcriptome in three subsequent generations of female offspring. Dams in the F1 and F2 generations were fed nutritionally adequate diets during their pregnancy. We also investigated for specific genes whether any differences in expression between offspring of F0 dams fed a protein-sufficient (PS) or PR diet during pregnancy were associated with altered promoter methylation.

## Results

Female Wistar rats were fed with an isocaloric PS or PR diet (n = 6 per dietary group) from conception until spontaneous delivery around day 21, then diet AIN93G during lactation. Female offspring were weaned onto AIN93M and either killed on day 70 or mated. F1 and F2 females were fed AIN93G throughout pregnancy and lactation, and F2 and F3 offspring were fed AIN93M from weaning. Livers were collected into liquid nitrogen on day 70, mRNA extracted and the expression of the transcriptome assessed by microarray using mRNA pooled from six female offspring in each generation. In F1 offspring, 1,684 genes (736 up-regulated, 948 down-regulated) differed by more than 2 fold between PS and PR lines. In F2 offspring, 1,680 genes (848 up-regulated, 832 down-regulated) differed between PS and PR lines. In F3 offspring, 23% more genes differed between PS and PR lines (total 2062 genes; 1,145 up-regulated; 917 down-regulated) compared to F1 and F2 offspring.

The twenty most up- or down- regulated genes in each generation are listed in Table 1. One gene, lymphocyte activation gene-3, was among the most up-regulated genes in all three generations. Pancreatic amylase was among the most up-regulated genes in F1 and the most down-regulated genes in F3, but was not among the twenty most altered genes in F2. Trefoil factor-2 was among the most up-regulated genes in F2 and F3, but was not among the twenty most altered genes in F1. No other genes were among the twenty most altered genes in more than one generation.

**Table 1:** Hepatic genes showing the greatest difference between PR and PS offspring.

Difference between PS and PR offspring (Fold differences between PR and PS)					
F1		F2		F3	
Up-regulated	Down-regulated	Up-regulated	Down-regulated	Up-regulated	Down-regulated
Cytochrome P450, 3,a NM_153312 (69.4)	Transmembrane protein 27 NM_020976 (174.3)	**Trefoil factor 2 NM_053844 (13.9)**	Olfactory receptor 144 NM_001000164 (15.3)	Surfactant associated protein C NM_017342 (25.1)	Pancreatic lipase related protein 1 NM_032081 (47.5)
α2μ globulin NM_001024248 (40.2)	Uromodulin NM_017082 (105.3)	Hyaluronan mediated motility receptor NM_012964 (12.9)	EGF-like domain 7 NM_139104 (14)	**Lymphocyte-activation gene 3 NM_212513 (17.4)**	Pancreatic lipase NM_013161 (39.6)
α2μ globulin PGCL4 NM_147215 (38.3)	Cytochrome P450 (11B) NM_012537 (104.9)	Sarcolipin NM_001013247 (10.0)	Type I keratin KA11 NM_001008750 (7.1)	Caveolin 3 NM_019155 (11.2)	**Pancreatic amylase 2 NM_031502 (39.4)**
**Lymphocyte-activation gene 3 NM_212513 (35.9)**	Chromogranin B NM_012526 (98.8)	Cathepsin R NM_001017509 (7.9)	T-cell receptor β-chain mRNA V-region M23885 (6.6)	Telethonin XM_001081394 (9.9)	Glycoprotein 2 NM_134418 (38.5)
α2μ globulin PGCL3 NM_147212 (29.1)	Chloride channel Kb NM_173103 (98.1)	Threonyl-tRNA synthetase-like 2 NM_001014020 (7.6)	Stathmin-like 3 NM_024346 (5.5)	Bile acid Coenzyme A: amino acid N-acyltransferaseXM_001055067 (9.7)	Cationic trypsinogen NM_173127 (34.4)
α2μ globulin PGCL1 NM_147214 (16.4)	Solute carrier family 7 (y+ system) member 13 NM_001012100 (92.6)	**Lymphocyte-activation gene 3 NM_212513 (7.5)**	Myelin-associated Basic Protein-99 X89638 (5.2)	RIKEN NM_001039686 (7.9)	Serine protease-2 NM_012729 (26.0)
Myosin IXb XM_346173 (15.2)	Defensin β1 NM_031810 (81.4)	C-myc promoter binding protein XR_009072 (7.3)	Cystatin NM_133566 (5.0)	Myosin, light polypeptide 3 NM_012606 (7.9)	Pancreatic trypsin 1 NM_012635 (24.8)
α2μ globulin PGCL5 NM_147213 (12.8)	Solute carrier family 26 (4) NM_019214 (80.1)	Gastrokine 1 XM_001074955 (6.4)	SOCS box-containing protein 3 XM_344277 (4.5)	Immunoglobulin variable region Z93370 (7.8)	Pancreatic lipase-related protein-2 NM_057206 (22.5)
Caldesmon 1 NM_013146 (12.3)	Protein phosphatase 1, regulatory subunit 1A NM_022676 (76.6)	Membrane-associated DHHC17 zinc finger protein NM_001039340 (5.8)	FUN14 domain containing-1 NM_001025027 (4.5)	Pancreatitis-associated protein NM_053289 (7.8)	Ribonuclease A NM_001029904 (19.8)
Major urinary protein 5 NM_203325 (11.3)	Solute carrier family 3 NM_017216 (71.0)	Unknown AF433878 (5.3)	Arachidonate 5-lipoxygenase NM_012822 (4.5)	Actin-α cardiac- 1 NM_019183 (7.7)	Chymotrypsin C NM_001077649 (19.1)
Cytochrome P450 (2c13) NM_138514 (10.4)	Kidney androgen regulated protein NM_052802 (67.6)	Glycosylation dependent cell adhesion molecule 1 NM_012794 (5.2)	Protocadherin-α NM_201422 (4.5)	EC1-V2R pheromone receptor XM_236825 (7.0)	Pancreatic elastase-1 NM_012552 (18.9)
α2μ globulin (L type) NM_001003409 (10.1)	Deoxyribonuclease 1 NM_013097 (60.8)	Actin α cardiac 1 NM_019183 (5.2)	Thioesterase superfamily member 4 (Them4), mRNA NM_001025017 (4.4)	S100 calcium binding protein G NM_012521 (7.0)	Chymotrypsin-like NM_054009 (17.0)
KPL2 protein NM_022620 (10.0)	Degenerative spermatocyte homolog 2 NM_001017457 (58.0)	Serine dehydratase X13119 (5.0)	Endothelial differentiation, sphingolipid G-protein-coupled receptor, 8 NM_021775 (4.3)	**Trefoil factor 2 NM_053844 (6.9)**	Carboxypeptidase B1 NM_012533 (16.5)
Cytochrome P450 (IIC) NM_019184 (8.1)	Solute carrier family 5 (sodium/glucose co-transporter), NM_022590 (57.6)	Phosphodiesterase 4D interacting protein NM_022382 (4.9)	Olfactory receptor 74 NM_001000134 (4.3)	Type I keratin KA15 NM_001004022 (6.9)	Trypsin 10 NM_001004097 (16.5)
Protein tyrosine kinase c-Yes XM_343644 (7.5)	Tonin NM_012677 (49.7)	Olfactory receptor 1250 NM_001000806 (4.8)	Olfactory receptor 821 NM_001000842 (4.3)	Topoisomerase -2α NM_022183 (6.7)	Chymotrypsinogen B NM_012536 (15.1)
Diamine acetyltransferase 1 XM_228448 (7.0)	Myosin, heavy polypeptide 7 NM_017240 (48.1)	PPARγ1 co-activator 1α NM_031347 (4.6)	KISS1 receptor NM_023992 (4.2)	Cleavage and polyadenylation specificity factor 3 XR_005746 (6.3)	Elastase 2 NM_012553 (11.9)
**Pancreatic amylase 2 NM_031502 (6.8)**	Cadherin 16 NM_001012055 (47.6)	Olfactory receptor 1467 NM_001000025 (4.6)	Calmodulin-sensitive plasma membrane Ca2+-transporting ATPase J05087 (4.2)	ATP-binding cassette (sub-family B, member 11) XM_579531 (6.2)	Pancreatic colipase NM_013139 (10.6)
Absent 1 isoform b XM_232591 (6.8)	Solute carrier family 7 (y+ system), member 12 NM_001017465 (47.1)	Cysteine and tyrosine-rich protein 1 NM_001013980 (4.6)	Seminal vesicle protein 4 NM_012662 (4.1)	Annexin A8 NM_001031654 (6.1)	Syncollin NM_139086 (9.4)
Glandular kallikrein 11 NM_001003977 (5.5)	Hydroxyacid oxidase-2 NM_032082 (45.1)	Neurogenic differentiation- 2 NM_019326 (4.6)	RT1 class Ib gene NM_012646 (4.1)	Olfactory receptor 272 NM_001000230 (6.0)	Serpin X99773 (9.2)
RAB3A NM_013018 (5.5)	Solute carrier family 12, member 1 NM_019134 (41.9)	RT1 class II, locus DOb NM_001008846 (4.3)	Dual oxidase 1 NM_153739 (4.1)	Lectin, galactose binding, soluble 4 NM_012975 (5.8)	Carboxyl ester lipase NM_016997 (9.2)

Genes which were among the twenty most different between PR and PS offspring in more than one generation are in bold font.

**Figure 1:**
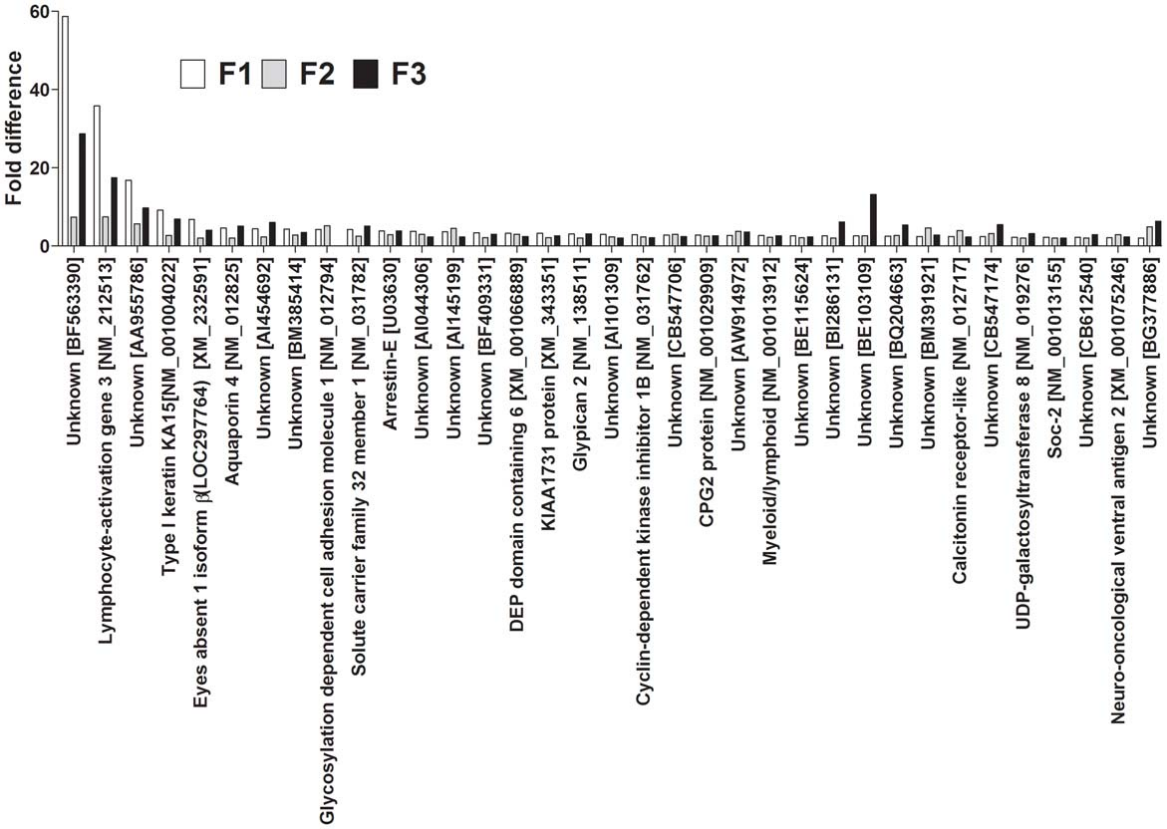
Genes up-regulated in PR compared to PS offspring lines in all three generations.

**Figure 2:**
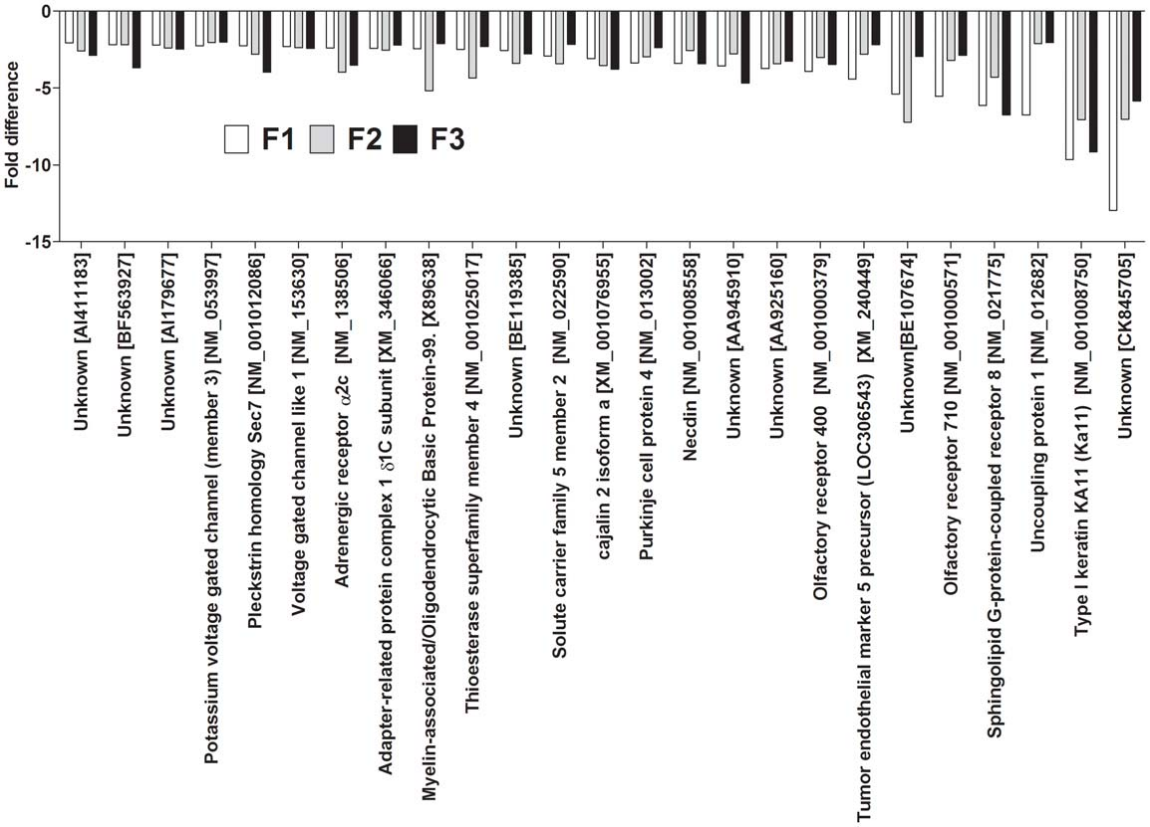
Genes down-regulated in PR compared to PS offspring lines in all three generations.

**Figure 3:**
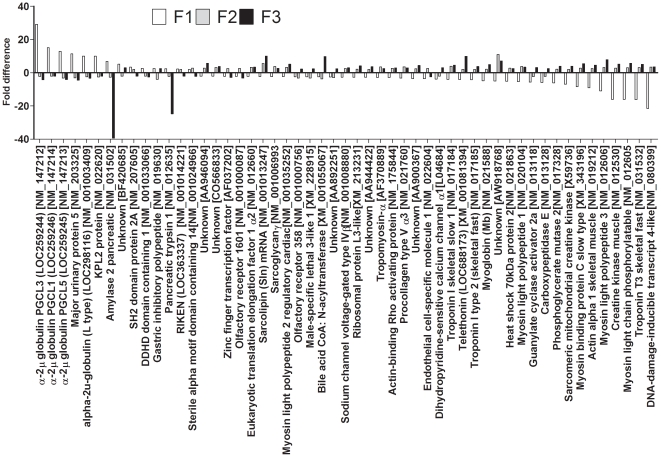
Genes which differed in the direction of difference between PR and Ps offspring across generations.

113 Genes differed by at least 2 fold between F0 dietary groups in all three generations. The number of up-regulated genes in F1 was 49/113, in F2 was 69/113 genes and in F3 was 71/113. 35/113 was up-regulated in all three generations (Figure 1) and 25/113 were down-regulated in all three generations in PR compared to PS offspring (Figure 2). However, 53/113 transcripts showed directionally opposite differences in expression between generations (Figure 3).

We validated the array by real time RTPCR for seven genes which differed in the patterns of difference between PS and PR offspring in samples from all three generations. Although the magnitude of difference between PS and PR offspring varied between real time RTPCR and the array, all seven genes showed the same direction of difference between PS and PR lines and the same pattern of change between generations when analysed either technique (Table 2). Linear regression analysis showed a significant positive association (P = 0.013, r = 0.86) between the difference between PR and PS samples in F3 detected by microarray and by real time RTPCR.

**Table 2:** Validation of microarray by real time RTPCR.

	Microarray			Real time RTPCR					
	Fold difference PR ∶ PS			Difference between means PR ∶ PS (%)			Student's t-test (P)		
Gene	F1	F2	F3	F1	F2	F3	F1	F2	F3
α-2μ Globulin PGCL3	12.8	2.2	−4.27	78	34	−42	0.006	0.0004	0.005
Major urinary protein-5	11.3	−3.1	−4.49	15	−11	−57	0.03	0.045	0.007
Myosin light chain polypeptide 3	−10.9	3.1	7.85	−22	18	30	0.005	0.009	0.004
Type 1 keratin KA11	−8.7	−7.1	−9.14	−31	−27	−49	0.002	0.007	0.02
Glycosylation-dependent cell adhesion molecular 1	4.3	5.2	−2.39	23	36	−22	0.02	0.008	0.01
Phosphofructokinase-2	−2.1	<2.0	2.20	−52	5	40	0.03	0.08	0.04
PEPCK	1.3	2.2	−2.0	66	84	−29	0.01	0.007	0.006

The direction of difference between offspring from the F0 PR and PS lines was assessed by micro array analysis of 6 pool samples and by real time RTPCR for the same six individual samples. Comparison of mRNA expression measured by RTPCR between PR and PS offspring was by Student's unpaired t-test.

**Figure 4:**
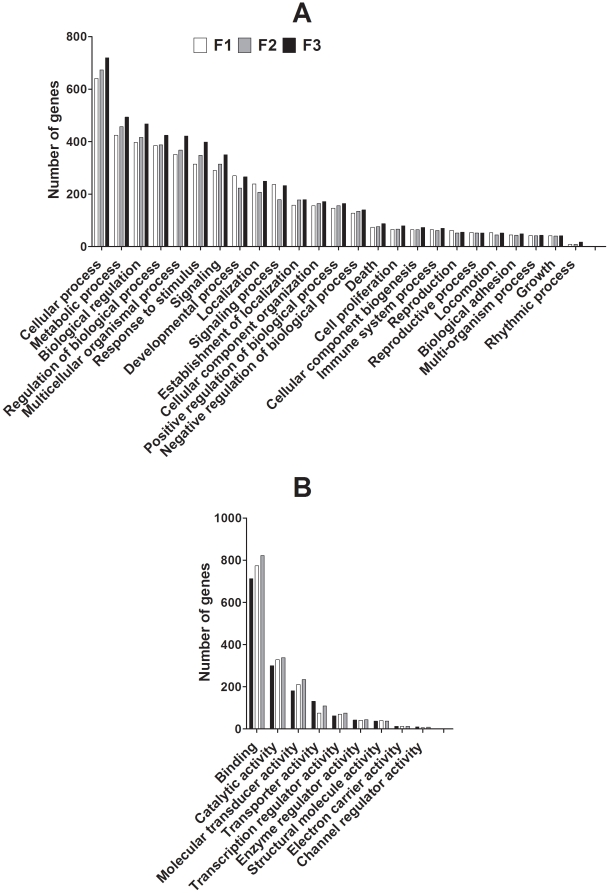
Number of genes which differed between PS and PR offspring in each generation according to ontology. Values are total numbers of genes which differed by at least two fold between PR and PS offspring in the (A) Biological Processes and (B) Molecular Function ontologies. The sub-ontologies indicated contained at least ten genes which differed between PR and PS offspring one or more generations. These data illustrate the number of genes which fall into each category rather than enrichment.

Assessment of the number of genes present in the Biological Processes and Molecular Functions ontologies showed differential changes within individual sub-ontologies. The data shown in Figure 4 do not indicate enrichment within individual categories. The total number of genes which showed differential expression between PS and PR offspring increased between F1, F2 and F3 generations in 15 of the 25 sub-ontologies of the Biological Processes ontology (Figure 4A). 3/25 showed a decrease in F2, but similar numbers in F1 and F3. The number of genes which differed by at least 2 fold between PR and PS offspring in the remaining ontologies was similar in all three generations. In the Molecular Functions ontology, 3/9 sub-ontologies showed an increase between F1 and F3 offspring in the number of genes which differed by at least 2 fold between PR and PS offspring (Figure 4B). One sub-ontology, Transporter Activity, showed a lower number of altered genes in F2 with a similar number of altered genes in F1 and F3. The remaining sub-ontologies showed similar numbers of altered genes in all three generations.

**Figure 5:**
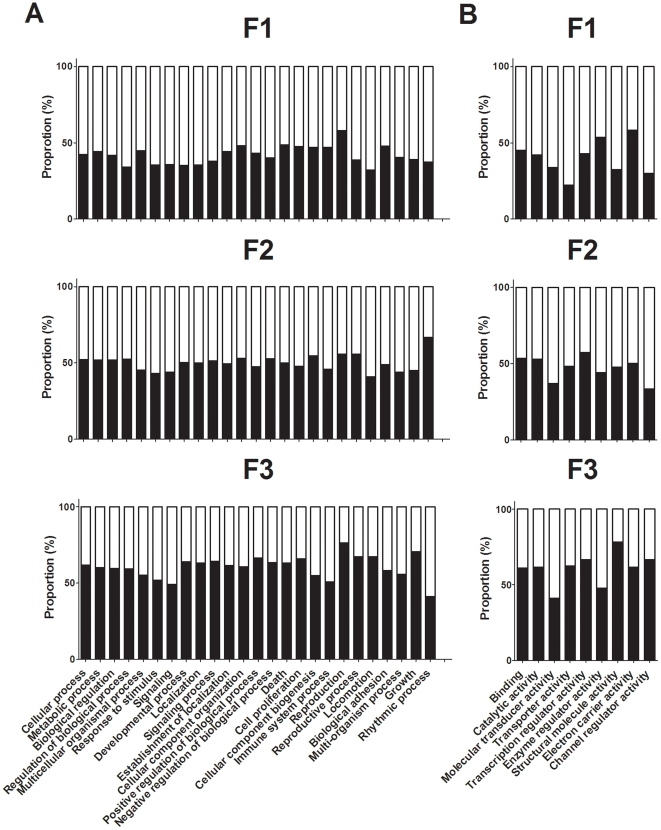
Proportion of genes which were up-regulated (solid bar) or down-regulated (open bar) in each generation. (A) Biological Processes ontology; (B) the Molecular Function ontology. The sub-ontologies indicated contained at least ten genes which differed between PR and PS offspring one or more generations. These data illustrate the proportions of genes showing either up or down regulation in each category rather than enrichment.

The data shown in Figure 5 show the proportions of genes within each category which were either up or down regulated, and do not indicate enrichment within categories. In the Biological Processes ontology, all but two of the sub ontologies, Reproductive Processes and Immune System Processes, showed an increase in the proportion of up-regulated genes in liver from F2 offspring compared to F1 (Figure 5A). In F3 offspring, the proportion of up-regulated genes was increased in all of the sub-ontologies compared to F1 offspring (Figure 5A). Compared to F2 offspring, 24/25 sub-ontologies showed an increase in up-regulated genes, while the proportion of down-regulated genes was increased in Rhythmic Processes, compared to F2 offspring (Figure 5A). In the Molecular Function ontology, the proportion of up-regulated genes was greater in F2 offspring in 7/9 sub-ontologies, while the proportion of up-regulated genes was decreased in Enzyme Regulator Activity and Electron Carrier Activity, compared to F1 offspring (Figure 5B). In F3 offspring, the proportion of up-regulated genes was greater in 8/9 sub-ontologies, but decreased in Enzyme Regulator Activity, compared to F1 offspring, but was increased in all sub-ontologies compared to F2 offspring (Figure 5B).

**Figure 6:**
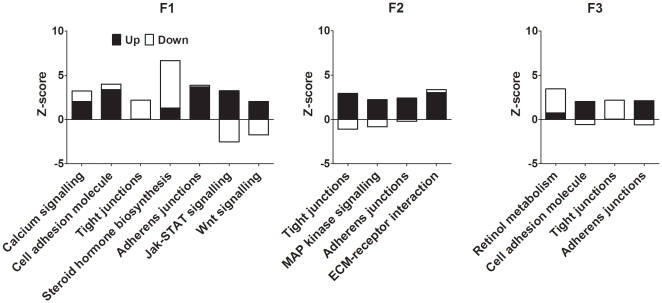
Canonical pathways involved in hepatic function which differed between PS and PR offspring. All pathways contained at least 10 genes which differed by at least 2 fold between offspring of PR and PS dams.

The results of KEGG analysis of pathways which are relevant to hepatic function which contained at least ten genes which differed by 2 fold or more between PS and PR lines are shown in Figure 6. In the F1 generation, Calcium Signalling, Cell Adhesion, Adherens Junction, Jak-STAT Signalling and Wnt Signalling pathways were over-represented amongst the up-regulated genes. Tight junction and Steroid Hormone Biosynthesis pathways were over-represented amongst the down-regulated genes, while Jak-STAT Signalling and Wnt Signalling pathways were under-represented amongst the down-regulated genes. In contrast to F1, the Tight Junctions pathway was over-represented amongst the up-regulated genes and under-represented amongst the down-regulated genes in F2. MAP Kinase Signalling and ECM Receptor Interaction pathways were over-represented amongst the up-regulated genes in F2, but were not altered in F1. In contrast to F2, but similar to F1, the Tight Junctions pathway was over-represented amongst the down-regulated genes in F3. Cell Adhesion Molecules and Retinol Metabolism pathways were altered in F3, but not F1 or F2. The Adherens Junction pathway was over-represented amongst the up-regulated genes in all three generations.

**Figure 7:**
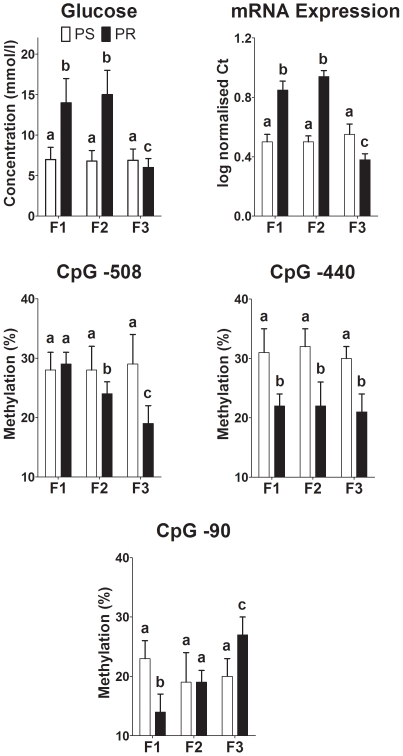
mRNA expression and methylation of specific CpG dinucleotides in the phosphoenolpyruvate carboxykinase promoter. CpGs are indicated by their location (bp) relative to the transcription start site. Values are mean ± SD of n = 6 samples per group per generation. Values significantly different (P<0.05) by a general linear model (GLM) with Bonferroni's *post hoc* test between generations are indicated by different letters. #Values significantly different between maternal dietary groups within a generation.

**Figure 8:**
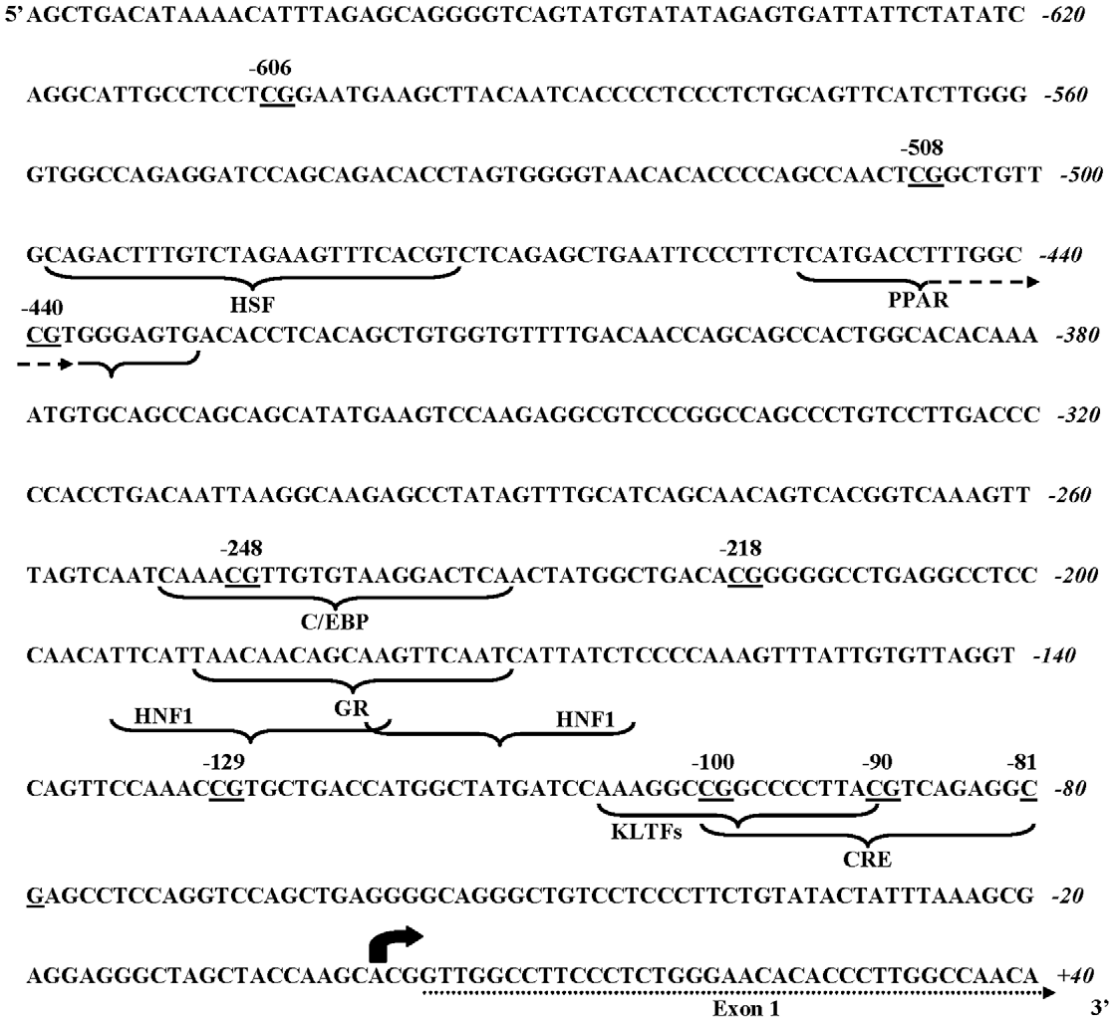
Genomic sequence of the region of the phosphoenolpyruvate carboxykinase promoter analysed for CpG methylation [**32**]. CpG reported in the methylation analysis are underlined. Know transcription factor response elements are indicated by curved brackets. HSF, heat shock factor; C/EBP, CATT enhancer-binding protein; HNF1, hepatic nuclear factor-1; KLTFs, Krueppel-like transcription factors; CRE, cAMP-response element.

Previous studies have shown that phosphoenolpyruvate carboxykinase (PEPCK) activity and mRNA expression increased in the offspring of dams fed a PR diet [21], [22]. PEPCK mRNA expression is regulated by the methylation status of its promoter [23]. Fasting plasma glucose was increased in F1 and F2, but was lower in F3, PR offspring compared to PS offspring (F0 diet, generation, interaction all P<0.0001) (Figure 7). We, therefore, measured the level of PEPCK mRNA expression and the methylation of individual CpG dinucleotides located within or proximal to response elements for transcription factors which are known to regulate PEPCK transcription [24] (Figure 8) in adult female offspring liver. There was a significant interaction between generation and F0 diet on PEPCK mRNA expression (P<0.0001) (Figure 7). There was no difference between generations in PEPCK expression in PS offspring. PEPCK expression was higher in PR offspring in F1 and F2, but lower in F3, than PS offspring. There was no significant effect of F0 diet on the methylation status of six of the nine CpGs which were measured in the PEPCK promoter (data not shown). However, CpG −508, was hypomethylated in F2 and F3 PR offspring compared to PS offspring (generation P = 0.009, F0 diet P<0.0001, interaction P = 0.002) (Figure 7). CpG −440 was hypomethylated in all three generations of PR offspring compared to PS offspring (generation not significant (NS); F0 diet P = 0.0023, interaction NS) (Figure 7). CpG −90 was hypomethylated in F1 PR offspring, did not differ significantly from PS offspring in F2, but was hypermethylated in F3 offspring compared to PS offspring (generation P<0.0001, F0 diet P = 0.015, interaction P = 0.022) (Figure 7).

## Discussion

The findings of this study show that expression of the liver transcriptome differed between offspring of F0 dams fed a PS or PR diet up to and including the third generation of offspring. Thus these data are in agreement with previous findings in the brain which showed that an insult during F0 pregnancy induced changes in the expression of the transcriptome for at least three generations [20]. Such observations are also consistent with reports of transgenerational effects of environmental challenge on the phenotype of at least the second generation offspring in humans [7], [8], insects [25], and in rodent experimental models [13], [26], [27].

The differences in the expression of the liver transcriptome between PS and PR lines differed between F1, F2 and F3 generations in terms of the number of genes which showed altered expression, the magnitude of difference, the distribution of up and down regulated genes and the ontologies affected. Furthermore, the number of genes which differed between PS and PR lines increased between F2 and F3 generations. Of the genes which differed between PS and PR offspring in all three generations, 47% differed between generations in the direction of the difference between PS and PR lines, while the remainder maintained the same direction of difference in all three generations. Together these findings do not support the suggestion that alterations in the transcriptome in F1 are passed without change to subsequent generations. Thus the expression of the transcriptome in the offspring of any generation following a challenge during F0 pregnancy cannot be assumed simply to reflect that of preceding or subsequent generations.

These findings are consistent with some reports which show variation or loss of induced phenotypes between generations. For example, feeding a PR diet during F0 pregnancy induced impaired glucose homeostasis in male F1 offspring which was exacerbated in F2, but then fell to a level below control offspring in F3 [13]. This is in contrast to stable prevention of inter-generational drift in body weight in offspring of F0 A^vy^ mice supplemented with methyl donors [28]. Together these findings suggest that the effects of altered nutrition on the phenotype of subsequent generations of offspring may be more complex than those mediated by a single exposure to an endocrine disruptor [18]. The present study showed that some genes, ontologies and pathways differed by a similar amount and in the same direction in each generation. These findings are consistent with those of Anway *et al.* [18], [19] and so suggest that for specific genes transmission of altered expression of may have occurred unchanged through the germline.

**Figure 9:**
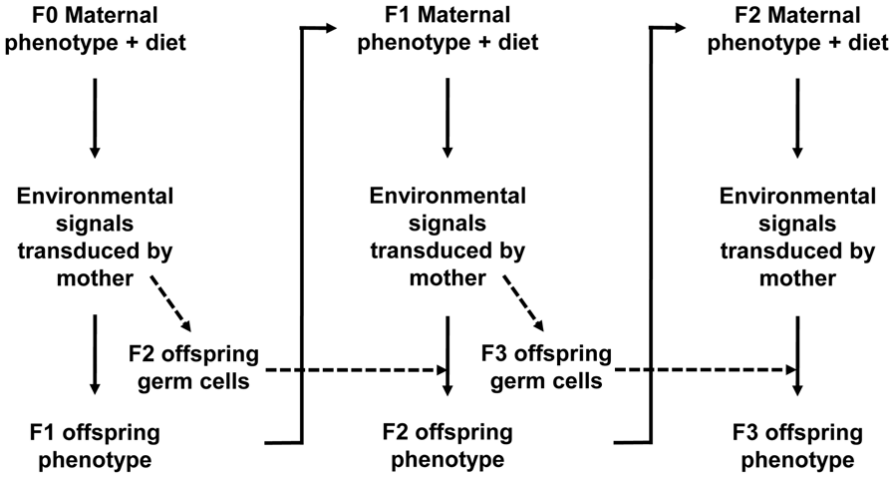
Model of transgenerational phenotypic variation.

The majority of genes which were altered in all three generations showed variation in the magnitude and direction of the effect of the maternal PR diet between generations. Transgenerational divergence in body weight has been shown between control A^vy^ mice and those supplemented with methyl donors, which implies an interaction between gene mutations and epigenetic processes [28], although the mechanism underlying the amplification of obesity is not known. One possible explanation is differences in the environment provided by the mother during development between generations providing different signals to the developing offspring in each generation (Figure 9). For example, the environmental signals received by the F1 fetus would reflect an interaction between the phenotype of the F0 dam with the PR diet leading to an altered phenotype in the F1 offspring. In turn, signals received by the F2 fetus would reflect the interaction of the phenotype of F1 dam with the environment, which differs from that of the F0 dam. The signals received by the F3 fetus would, therefore, reflect an interaction between the phenotype of the F2 dam and the environment, which differs from those of the F0 and F1 dams. Furthermore, germ cells destined to become F2 offspring would be exposed to signals from the F0 dams and those from the F1 dams, and those destined to become F3 offspring would be exposed to signals from the F1 dam as wells the F2 dam. Together, such complex interactions between mother and offspring may provide a mechanism by which the number of altered genes is greater in F3 than F1, and by which ontologies and pathways are altered in F3, but not in previous generations. Such maternal effects on the phenotype of the offspring are well-established and have been suggested to be a mechanism for generation of novel phenotypes [29], although the molecular basis of such changes has not been shown previously. This model also suggests a mechanism by which single phenotypic traits induced in F1 offspring are apparently lost in future generations [14] and so emphasises a need for comprehensive analysis of the phenotype of offspring in transgenerational studies.

The twenty genes which showed the greatest difference between PR and PS offspring in each generation had diverse function, some of which, for example surfactant protein C, have uncertain functional significance in liver. Two of the genes which differed between PR and PS offspring in more than one generation, trefoil factor-2 and lymphocyte activation gene-3, are associated with tissue repair and limitation of immune response, respectively, although whether they exhibit these function in liver is unclear. Amylase-2 activity has been demonstrated in liver, although its contribution of glycogenolysis relative to glycogen phosphorylase is unclear [30]. Amyase-2 was up-regulated in F1 PR offspring which had raised fasting blood glucose concentration but was down-regulated in F3 PR offspring with normal blood glucose. This suggests that altered hepatic amylase-2 activity may contribute to differences between generations in glucose homeostasis between PS and PR offspring.

Fasting plasma glucose was increased in F1 and F2, but not F3 offspring. Because gluconeogenesis is the major source of plasma glucose in rats fasted for 12 hours [31] and F1 offspring of dams fed a PR diet show increased gluconeogenic activity [22], we measured the mRNA expression and methylation of the PEPCK promoter in the liver of the offspring. The pattern of PEPCK mRNA expression between generations followed that of plasma glucose concentration, with the exception that PEPCK expression was lower in PR than PS offspring in F3. This suggests that, in agreement with previous findings of the effect of prenatal exposure of F1 offspring to dexamethasone [14], changes in the regulation of PEPCK transcription are an important process underlying differences in plasma glucose concentrations between generations. Of the nine CpGs measured in the PEPCK promoter, three CPGs showed differential methylation between PS and PR lines. All three are located within known transcription factor response elements [32] and thus changes in the level of methylation may be expected to be associated with altered transcriptional activity. CpG −508 is located proximal to a heat-shock factor response element, while CpG −440 is within a PPAR response element and CpG −90 within a cAMP response element. The PEPCK promoter contains multiple response elements and hence overall capacity for transcription represents the overall regulation of its activity. Thus the level of PEPCK mRNA cannot be assumed to simply reflect the methylation status of single CpGs. However, CpGs −508, −440 and −90 each showed specific changes between generations in the relative level of methylation between PS and PR offspring. These differences in the methylation of individual CpGs are consistent with the models described above for the transmission of induced phenotypes between generations. Thus methylation of CpG −440 may be transmitted unchanged through germ cells, although this would require the preservation of these epigenetic marks through, albeit incomplete, genome-wide demethylation at fertilisation [33]. In contrast, the patterns of change in CpGs −508 are consistent with differences in maternal signals between generations. Thus these findings support the suggestion that epigenetic processes may be involved in the transmission of induced phenotypes between generations.

Transmission of phenotypic changes induced during their development may represent an important source of variation in disease risk in subsequent generations [7], [8]. The present findings suggest that such transmission of phenotypes involves adjustments in the expression of the transcriptome and in underlying epigenetic processes between generations. If replicated in humans, identification of such processes may provide targets for therapeutic interventions and tools for assessing their effectiveness. However, these observations also suggest that multiple processes may be involved in the passage of induced phenotypes and epigenotype between generations. This potentially represents a challenge to the development of interventions to prevent the passage of such effects between generations.

## Materials and Methods

### Ethical statement

The study was carried out in accordance with the United Kingdom Home Office Animals (Scientific Procedures) Act (1986) and was conducted under Home Office Licence number 70-6457.

### Animals and tissues

Female Wistar rats (about 220 g) obtained from a breeding colony were maintained on standard chow for 14 days and then mated. No male was mated with any of its progeny. F0 Dams were fed either a PS or PR diet (n = 6 per dietary group) during pregnancy which provided an increase in energy of approximately 25% compared to the diet fed to the breeding colony (Table 3). Dams were fed AIN93G during lactation and offspring were weaned onto AIN93M on postnatal day 28. Litters were standardised to 8 offspring within 24 hours of birth, with bias towards females to ensure sufficient stock for mating. F1 and F2 females were mated on postnatal day 70 (n = 6 per F0 dietary group). F1 and F2 dams were fed the PS diet during pregnancy and AIN93G during lactation. Offspring were weaned onto AIN93M. All female offspring which were not mated were fasted for 12 hours (20:00 to 08:00) and then killed by carbon dioxide asphyxiation on postnatal day 70. Livers were frozen immediately in liquid nitrogen. Blood was collected into heparinised tubes. Plasma was separated by centrifugation and stored at −80°C.

**Table 3:** Diet composition.

	Pregnancy diets		Lactation diet	Maintenance diet
	PS (all generations)	PR (F0 generation)	AIN-93G	AIN-93M
Casein (g/kg)	18.3	92	200	140
Cornstarch (g/kg)	420	482	397	466
Sucrose (g/kg)	213	243	100	100
Choline (g/kg)	2.8	2.8	2.5	2.5
Methionine (g/kg)	9.7	7.4	5.2	3.6
Crude fibre (g/kg)	50	50	50	50
Oil (g/kg)	100	100	70	40
Total metabolisable energy (MJ/kg)	17.2	17.4	16.4	15.78

### Measurement of fasting plasma glucose concentration

Plasma glucose concentration was measured by an automated colorimetric method as described [34].

### RNA isolation and measurement of the expression of the liver transcription by Agilent oligonucleotide array hybridisation

RNA was extracted from livers from PS and PR offspring (n = 6 per F0 dietary group in each generation) using TRIzol reagent (Invitrogen) according to manufacturer's instructions. RNA was quantified by absorbance at 260 nm, and the integrity of the 28 s and 18 s ribosomal RNA was verified by agarose gel electrophoresis. In all cases the absorbance ratio at 260 and 280 nm was greater than 2. Equal amounts (300 ng) of RNA from each group of six offspring were pooled and this preparation was used for microarray analysis using the Two Colour Microarray Based Gene expression analysis (Agilent Technologies, Inc., Palo Alto, CA). Microarray hybridisation and analysis was carried out as described [35] by Oxford Gene Technology (OGT, Oxford UK) in accordance with the company's quality control procedures using standard protocols for labelling, hybridisation and washing. RNA was reverse transcribed into cDNA. After denaturation of the reverse transcriptase enzyme, samples were transcribed into cRNA and labelled with the fluorescent dye Cy (test sample Cy3, reference sample Cy5) and was hybridised to an Agilent 014879 whole rat genome array (4×44 K) G4131F. This array contains 45,018 features with 41,012 unique probes. Microarray slides were scanned at 5 µM resolution using the extended dynamic range (Hi 100%, Low 10%). The slides were feature extracted using Agilent feature extraction software 9.5.3.1. The results were uploaded into Genespring GX V 7.3 (Silicon Graphics Inc) for data normalisation, quality control and first pass analysis. All arrays were adjusted for within slide intensity dependent variation due to dye properties Lowess normalisation using Genespring (http://stat-www.berkeley.edu/users/terry/zarray/Html/normspie.html). The expression ratios were calculated for each probe by dividing the Cy3 processed signal by Cy5 processed signal. The identification of the genes showing increased or decreased expression was performed using GeneSifter™ software (www.genesifter.net; VizX Labs LLC, Seattle, WA, USA). Only transcripts which differed by at least 2 fold between PS and PR offspring were considered to be changed. All data are MIAME compliant and the raw data are deposited at www.ebi.ac.uk/arrayexpress, accession number E-MEXP-3205.

### Real time RTPCR

Real time RTPCR was carried out essentially as described [36]. mRNA expression of hepatic genes was measured by real-time PCR. Briefly, total RNA was isolated from cells with TRIzol reagent (Invitrogen, Paisley, Scotland, U.K.), and 1 µg was used as a template to prepare cDNA with 100 units of Moloney murine leukemia virus reverse transcriptase. cDNA was amplified with real-time PCR primers (Table 4). The reaction was performed in a total volume of 25 µl with SYBR Green Jumpstart Ready Mix (Sigma, Poole, Dorset, U.K.) as described by the manufacturer. Samples were analyzed in duplicate, and Ct values were normalized to cyclophilin [36].

**Table 4:** Real time RTPCR primers.

	Real time RTPCR	
	Forward Primer (5′→3′)	Reverse Primer (3′→5′)
Gene	mRNA expression	
Cyclophilin	TTGGGTCGCGTCTGCTTCGA	GCCAGGACCTGTATGCTTCA
PEPCK	AGCTGCATAATGGTCTGG	GAACCTGGCGTTGAATGC
Glycosylation-dependent cell adhesion molecular 1	Quantitect primer assay QT00185934	
Phosphofructokinase-2/Fructose-2,6- bisphosphatase	Quantitect primer assay QT00185395	
Myosin light chain polypeptide 3	Quantitect primer assay QT00193648	
α-2μ Globulin PGCL5	Quantitect primer assay QT00195545	
Major urinary protein 5	Quantitect primer assay QT01791104	
Type I keratin/Ka11	Quantitect primer assay QT01818747	

### Analysis of PEPCK promoter methylation by pyrosequecing

The level of methylation of individual CpG dinucleotides was measured in a region between 89 and 606 bp upstream from the transcription start site which had known regulatory function [24], [32] essentially as described [37]. Briefly, genomic DNA was prepared and bisulphite conversion was carried out using the EZ DNA methylation kit (ZymoResearch). The pyrosequencing reaction was carried out using primers listed in Table 5. Modified DNA was amplified using KAPA2G Fast HotStart DNA polymerase (Kapa Biosystems). PCR products were immobilised on streptavidin–sepharose beads (Amersham), washed, denatured and released into annealing buffer containing the sequencing primers (Table 5). Pyrosequencing was carried out using the SQA kit on a PSQ 96MA machine (Biotage) and the percentage methylation was calculated using the Pyro Q CpG (Biotage). Within assay precision was between 0·8 and 1·7% depending on CpG, and detection limits were 2–5% methylation.

**Table 5:** Pyrosequencing primers and phosphoenolpyruvate carboxykinase CpG identifiers.

	Real time RTPCR	
Primer location (bp relative to transcription start site)	Forward Primer (5′→3′)	Reverse Primer (3′→5′)
	PCR primers	
−658 to −405	AGGGGTTAGTATGTATATAGAGTGATT	ATCAAAACACCACAACTATAAAATATC
−417 to −56	GTGGTGTTTTGATAATTAGTAGTGATT	CCCCTCAACTAAACCTAAAAACTC
−373 to −44	GTTAGTAGTATATGAAGTTTAAGA	CCCCTATTAACCAAAAATATATTCC
−658 to −405	AGGGGTTAGTATGTATATAGAGTGATT	ATCAAAACACCACAACTATAAAATATC
−417 to −56	GTGGTGTTTTGATAATTAGTAGTGATT	CCCCTCAACTAAACCTAAAAACTC
−373 to −44	GTTAGTAGTATATGAAGTTTAAGA	CCCCTATTAACCAAAAATATATTCC
	
	Sequencing primers	
	GTGATTATTTTATATTAGGTATTG	
	AGAGGATTTAGTAGATATTTAGTG	
	TAAATATTAAAAAACCTCAAACCC	
	TTATTATTTTTTTAAAGTTTATTG	
	
	CpG locations	
	CpG Location relative to transcription start site (bp)	Chromosome 3 coordinate (bp)
	−606	164,012,404
	−508	164,012,502
	−440	164,012,570
	−248	164,012,762
	−218	164,012,792
	−129	164,012,881
	−100	164,012,910
	−90	164,012,920
	−81	164,012,929

### Statistical analysis

For analysis of glucose concentration, and PEPCK methylation and expression, values are shown as mean ± 1 SD. Comparison between groups and between generations of single factor and interactive effects on glucose concentration, and PEPCK methylation and expression were by a general linear model with F0 diet and generation as fixed factors, and Bonferroni's *post hoc* test. The results of real time RTPCR analysis were non-parametric and were log_10_ transformed before statistical analysis.

## References

[1] Gluckman PD, Hanson MA, Cooper C, Thornburg KL (2008). Effect of in utero and early-life conditions on adult health and disease.. N Engl J Med.

[2] Bertram CE, Hanson MA (2001). Animal models and programming of the metabolic syndrome.. Br Med Bull.

[3] Gluckman PD, Hanson MA (2004). Living with the past: evolution, development, and patterns of disease.. Science.

[4] Jablonka E, Raz G (2009). Transgenerational Epigenetic Inheritance: Prevalence, Mechanisms, and Implications for the Study of Heredity and Evolution.. Quart Rev Biol.

[5] Gluckman PD, Hanson MA (2005). Metabolic disease: evolutionary, developmental and transgenerational influences.. Nestle Nutr Workshop Ser Pediatr Program.

[6] Gluckman PD, Hanson MA, Beedle AS (2007). Non-genomic transgenerational inheritance of disease risk.. Bioessays.

[7] Pembrey ME, Bygren LO, Kaati G, Edvinsson S, Northstone K (2006). Sex-specific, male-line transgenerational responses in humans.. Eur J Hum Genet.

[8] Painter RC, Osmond C, Gluckman P, Hanson M, Phillips DI (2008). Transgenerational effects of prenatal exposure to the Dutch famine on neonatal adiposity and health in later life.. BJOG.

[9] Stein AD, Lumey LH (2000). The relationship between maternal and offspring birth weights after maternal prenatal famine exposure: the Dutch Famine Birth Cohort Study.. Hum Biol.

[10] Torrens C, Poston L, Hanson MA (2008). Transmission of raised blood pressure and endothelial dysfunction to the F2 generation induced by maternal protein restriction in the F0, in the absence of dietary challenge in the F1 generation.. Br J Nutr.

[11] Martin JF, Johnston CS, Han CT, Benyshek DC (2000). Nutritional origins of insulin resistance: A rat model for diabetes-prone human populations.. J Nutr.

[12] Zambrano E, Martinez-Samayoa PM, Bautista CJ, Deas M, Guillen L (2005). Sex differences in transgenerational alterations of growth and metabolism in progeny (F-2) of female offspring (F-1) of rats fed a low protein diet during pregnancy and lactation.. J Physiol.

[13] Benyshek DC, Johnston CS, Martin JF (2006). Glucose metabolism is altered in the adequately-nourished grand-offspring (F-3 generation) of rats malnourished during gestation and perinatal life.. Diabetologia.

[14] Drake AJ, Walker BR, Seckl JR (2005). Intergenerational consequences of fetal programming by in utero exposure to glucocorticoids in rats.. Am J Physiol Regul Integr Comp Physiol.

[15] Cropley JE, Suter CM, Beckman KB, Martin DI (2006). Germ-line epigenetic modification of the murine A^vy^ allele by nutritional supplementation.. Proc Natl Acad Sci USA.

[16] Burdge GC, Slater-Jefferies J, Torrens C, Phillips ES, Hanson MA (2007). Dietary protein restriction of pregnant rats in the F0 generation induces altered methylation of hepatic gene promoters in the adult male offspring in the F1 and F2 generations.. Br J Nutr.

[17] Skinner MK (2008). What is an epigenetic transgenerational phenotype? F3 or F2.. Reprod Toxicol.

[18] Anway MD, Cupp AS, Uzumcu M, Skinner MK (2005). Epigenetic transgenerational actions of endocrine disruptors and male fertility.. Science.

[19] Anway MD, Rekow SS, Skinner MK (2008). Transgenerational epigenetic programming of the embryonic testis transcriptome.. Genomics.

[20] Skinner MK, Anway MD, Savenkova MI, Gore AC, Crews D (2008). Transgenerational epigenetic programming of the brain transcriptome and anxiety behavior.. PLoS ONE.

[21] Burdge GC, Lillycrop KA, Jackson AA, Gluckman PD, Hanson MA (2008). The nature of the growth pattern and of the metabolic response to fasting in the rat are dependent upon the dietary protein and folic acid intakes of their pregnant dams and post-weaning fat consumption.. Br J Nutr.

[22] Burns SP, Desai M, Cohen RD, Hales CN, Iles RA (1997). Gluconeogenesis, Glucose Handling, and Structural Changes in Livers of the Adult Offspring of Rats Partially Deprived of Protein During Pregnancy and Lactation.. J Clin Invest.

[23] Benvenisty N, Szyf M, Mencher D, Razin A, Reshef L (1985). Tissue-specific hypomethylation and expression of rat phosphoenolpyruvate carboxykinase gene induced by in vivo treatment of fetuses and neonates with 5-azacytidine.. Biochem.

[24] Yang J, Reshef L, Cassuto H, Aleman G, Hanson RW (2009). Aspects of the control of phosphoenolpyruvate carboxykinase gene transcription.. J Biol Chem.

[25] Mousseau TA, Dingle H (1991). Maternal Effects in Insect Life Histories.. Annual Review of Entomology.

[26] Chandra RK (1975). Antibody-Formation in First and 2Nd Generation Offspring of Nutritionally Deprived Rats.. Science.

[27] Zamenhof S, Vanmarth E, Grauel L (1971). DNA (Cell Number) in Neonatal Brain - Second Generation (F2) Alteration by Maternal (F0) Dietary Protein Restriction.. Science.

[28] Waterland RA, Travisano M, Tahiliani KG, Rached MT, Mirza S (2008). Methyl donor supplementation prevents transgenerational amplification of obesity.. Int J Obes (Lond).

[29] Badyaev AV (2008). Maternal effects as generators of evolutionary change: a reassessment.. Ann N Y Acad Sci.

[30] Koyama I, Komine S, Hokari S, Yakushijin M, Matsunaga T (2001). Expression of alpha-amylase gene in rat liver: liver-specific amylase has a high affinity to glycogen.. Electrophoresis.

[31] Herrera E, Freinkel N (1968). Interrelationships between liver composition, plasma glucose and ketones, and hepatic acetyl-CoA and citric acid during prolonged starvation in the male rat.. Biochim Biophys Acta.

[32] Beale EG, Chrapkiewicz NB, Scoble HA, Metz RJ, Quick DP (1985). Rat hepatic cytosolic phosphoenolpyruvate carboxykinase (GTP). Structures of the protein, messenger RNA, and gene.. J Biol Chem.

[33] Bird A (2002). DNA methylation patterns and epigenetic memory.. Genes Dev.

[34] Burdge GC, Sala-Vila A, West AL, Robson HJL, Le Fevre LW (2007). The effect of altering the 20:5n-3 and 22:6n-3 content of a meal on the postprandial incorporation of n-3 polyunsaturated fatty acids into plasma triacylglycerol and non-esterified fatty acids in humans.. Prost Leuk Essent Fatty Acids.

[35] Lillycrop KA, Rodford J, Garratt ES, Slater-Jefferies JL, Godfrey KM (2010). Maternal protein restriction with or without folic acid supplementation during pregnancy alters the hepatic transcriptome in adult male rats.. Br J Nutr.

[36] Burdge GC, Lillycrop KA, Phillips ES, Slater-Jefferies JL, Jackson AA (2009). Folic Acid Supplementation during the Juvenile-Pubertal Period in Rats Modifies the Phenotype and Epigenotype Induced by Prenatal Nutrition.. J Nutr.

[37] Lillycrop KA, Phillips ES, Torrens C, Hanson MA, Jackson AA (2008). Feeding pregnant rats a protein-restricted diet persistently alters the methylation of specific cytosines in the hepatic PPARalpha promoter of the offspring.. British Journal of Nutrition.

